# Protein Disulfide Isomerase Interacts with Tau Protein and Inhibits Its Fibrillization

**DOI:** 10.1371/journal.pone.0076657

**Published:** 2013-10-02

**Authors:** Li-Rong Xu, Xiao-Ling Liu, Jie Chen, Yi Liang

**Affiliations:** State Key Laboratory of Virology, College of Life Sciences, Wuhan University, Wuhan, China; University of Akron, United States of America

## Abstract

**Background:**

Tau protein is implicated in the pathogenesis of neurodegenerative disorders such as tauopathies including Alzheimer disease, and Tau fibrillization is thought to be related to neuronal toxicity. Physiological inhibitors of Tau fibrillization hold promise for developing new strategies for treatment of Alzheimer disease. Because protein disulfide isomerase (PDI) is both an enzyme and a chaperone, and implicated in neuroprotection against Alzheimer disease, we want to know whether PDI can prevent Tau fibrillization. In this study, we have investigated the interaction between PDI and Tau protein and the effect of PDI on Tau fibrillization.

**Methodology/Principal Findings:**

As evidenced by co-immunoprecipitation and confocal laser scanning microscopy, human PDI interacts and co-locates with some endogenous human Tau on the endoplasmic reticulum of undifferentiated SH-SY5Y neuroblastoma cells. The results from isothermal titration calorimetry show that one full-length human PDI binds to one full-length human Tau (or human Tau fragment Tau_244–372_) monomer with moderate, micromolar affinity at physiological pH and near physiological ionic strength. As revealed by thioflavin T binding assays, Sarkosyl-insoluble SDS-PAGE, and transmission electron microscopy, full-length human PDI remarkably inhibits both steps of nucleation and elongation of Tau_244–372_ fibrillization in a concentration-dependent manner. Furthermore, we find that two molecules of the a-domain of human PDI interact with one Tau_244–372_ molecule with sub-micromolar affinity, and inhibit both steps of nucleation and elongation of Tau_244–372_ fibrillization more strongly than full-length human PDI.

**Conclusions/Significance:**

We demonstrate for the first time that human PDI binds to Tau protein mainly through its thioredoxin-like catalytic domain a, forming a 1∶1 complex and preventing Tau misfolding. Our findings suggest that PDI could act as a physiological inhibitor of Tau fibrillization, and have applications for developing novel strategies for treatment and early diagnosis of Alzheimer disease.

## Introduction

Tau protein is implicated in the pathogenesis of neurodegenerative disorders such as tauopathies including Alzheimer disease, and Tau fibrillization is thought to be related to neuronal toxicity [1]–[3]. There is no efficient treatment available for Alzheimer disease so far, and the mechanism of Alzheimer disease is still unclear [4], [5]. Thus on one hand, the characterization of factors regulating Tau fibrillization is of great importance to clarify the etiology of Alzheimer disease and to assist in the establishment of medical treatment [4], [5]. On the other hand, physiological inhibitors for Tau fibrillization, such as molecular chaperone heat shock protein 70 (Hsp70) [6], [7], hold promise for development of novel strategies for treatment and early diagnosis of Alzheimer disease.

Tau protein does not adopt the compact folded structure typical of most cytosolic proteins because of its hydrophilic character [8]. Instead, Tau protein adopts a natively unfolded or intrinsically disordered structure in solution [9], [10]. Tau protein consists of two distinct domains, the projection domain and the microtubule binding domain, and four or three imperfect repeats (R1, R2, R3, and R4) make up the microtubule binding domain [10], [11]. Tau_244–372_ contains the four-repeat microtubule binding domain forming the core of bundles of filaments in Alzheimer disease, and can form fibrils with the help of heparin *in vitro* in a relatively short time [4], [12]–[15]. Thus it is a frequently used model for Tau fibrillization [16].

The endoplasmic reticulum (ER), a central player in cell physiology, not only is an important organelle for protein folding and protein posttranslational modification, but also acts as a significant intracellular calcium store [17]. The accumulation of misfolded proteins and Ca^2+^ influx can cause ER stress in neurons, and severe ER stress can induce apoptosis [18], [19]. However, the ER can withstand relatively mild insults through the expression of molecular chaperones such as Hsp70 and protein disulfide isomerase (PDI) [20].

PDI is both an enzyme and a chaperone [21] primarily located at the ER [22], and implicated in neuroprotection against multiple neurodegenerative diseases including Alzheimer disease [23]. PDI is composed of four thioredoxin domains a, b, b’, and a’, an x-linker between b’ and a’, and a C-terminal acidic tail [24]–[27]. Both the a and a’ domains are thioredoxin-like catalytic domains containing a CGHC active site, and are capable of catalyzing simple oxidoreductions and thiol-disulfide exchange reactions [28]. The b’ domain provides the main peptide binding site. When PDI catalyzes a complex disulfide isomerization associated with protein folding (involves both thiol/disulfide chemistry and substantive conformational change in the substrate), all the four domains of PDI are required to function synergistically, combining the chaperone activity with the foldase activity [27], [29]–[31].

As an important chaperone induced by ER stress, PDI is believed to accelerate the folding of disulfide-bonded proteins by catalyzing the disulfide interchange reaction [22]. Recently, multiple neurodegenerative diseases including Alzheimer disease are shown to be associated with an upregulation of PDI expression level [20], [23], [32], [33]. Furthermore, PDI and paired helical filament-Tau are co-located in neurofibrillary tangles in the brain of patients with Alzheimer disease [34], [35]. Because PDI is both an enzyme and a chaperone, and implicated in neuroprotection against Alzheimer disease as described above, we want to know whether PDI can prevent Tau fibrillization.

In the present study, by using co-immunoprecipitation and confocal laser scanning microscopy and several biophysical methods, such as thioflavin T (ThT) binding assays, isothermal titration calorimetry (ITC), Sarkosyl-insoluble SDS-PAGE, and transmission electron microscopy (TEM), we investigated the interaction of full-length human PDI with human Tau protein as well as the effect of human PDI on the fibrillization of human Tau fragment Tau_244–372_ under physiological conditions. We found that PDI interacted with some endogenous Tau protein in the cell lines and showed co-localization in the ER. Our biophysical analysis revealed that one full-length PDI bound to one full-length Tau monomer with micromolar affinity at physiological pH. We also found that PDI (especially thioredoxin-like catalytic domain a) inhibited nucleation and elongation of Tau_244–372_ fibrillization. We thus suggest that PDI could act as a physiological inhibitor of Tau fibrillization, protecting ER from the harmful effect of misfolded Tau protein. Our findings can be used to develop novel strategies for treatment of Alzheimer disease.

## Materials and Methods

### Ethics statement

All research involving original human work was approved by the Institutional Review Board of the College of Life Sciences, Wuhan University (Wuhan, China), leaded by Dr. Hong-Bing Shu, the Dean of the college, in accordance with the guidelines for the protection of human subjects. Written informed consent for the original human work that produced the plasmid samples was obtained.

### Materials

Heparin (average MW = 6 kDa) and ThT were purchased from Sigma-Aldrich (St. Louis, MO). The crowding agent Ficoll 70 was also obtained from Sigma-Aldrich. Dithiothreitol (DTT), Sarkosyl and sodium dodecyl sulfate (SDS) were purchased from Amresco (Solon, OH). Ni Sepharose™ high performance was obtained from GE Healthcare (Uppsala, Sweden), and SP Sepharose Fast Flow was Amersham Biosciences products (Uppsala, Sweden). All other chemicals used were made in China and were of analytical grade.

### Plasmids and proteins

All of the recombinant human PDI proteins (full-length human PDI and its fragments abb’xa’, bb’x, a’c and a) plasmids were kindly provided by Prof. Lloyd W. Ruddock (Department of Biochemistry, University of Oulu, Oulu, Finland). The cDNA encoding human Tau fragment Tau_244–372_ was amplified using the plasmid for human Tau40 (kindly provided by Dr. Michel Goedert) as a template. The PCR-amplified Tau_244–372_ was subcloned into pRK172 vector. The sequences of all the constructs were verified by DNA sequencing. Recombinant full-length human Tau and Tau_244–372_ were expressed in *Escherichia coli* and purified to homogeneity by SP-Sepharose chromatography as described [4], [36]. Purified Tau protein was analyzed by SDS-PAGE with one band and confirmed by mass spectrometry. The concentrations of full-length human Tau and Tau_244–372_ were determined according to their absorbance at 214 nm with a standard curve drawn by bovine serum albumin.

Recombinant full-length human PDI and its fragments were expressed in *Escherichia coli* BL21 (DE3) pLysS strain and purified to homogeneity by Ni Sepharose chromatography as described [37]. The resulting proteins all included an N-terminal His tag (MHHHHHHM). Protein concentrations of full-length human PDI and its fragments were determined by the Bradford method with bovine serum albumin as a standard.

### Cell culture and transfection

SH-SY5Y neuroblastoma cells were cultured in Dulbecco's modified Eagle's medium supplemented with 10% (v/v) fetal bovine serum (FBS), 100 U/ml penicillin, and 100 U/ml streptomycin in 5% CO_2_ at 37°C. SH-SY5Y cells were transiently transfected with HA-tagged full-length human PDI in pcDNA 3.1 vector using Lipofectamine® 2000 (Invitrogen, Carlsbad, CA) according to the manufacturer's protocol and harvested 36 h after transfection.

### Co-immunoprecipitation (Co-IP) assays

Approximately 36 h after cell transfection, SH-SY5Y cells were rinsed three times in ice-cold PBS buffer, followed by incubation with 1 ml of ice-cold IP lysis buffer (Beyotime, Haimen, China) containing a serine protease inhibitor PMSF for 30 min at 4°C. Samples were pre-cleared with Protein A+G agarose (Beyotime). Pre-cleared lysates were then incubated with mouse monoclonal anti-HA antibody (Sigma-Aldrich) at 4°C overnight. A 25% slurry of Protein A+G agarose was added into the lysates, incubated for 2 h at 4°C, and washed with ice-cold IP lysis buffer (Beyotime). The pellet was re-suspended in SDS loading buffer, boiled for 10 min, and then centrifugated at 17,000 g for 1 min with an Eppendorf 5810R centrifuge (Eppendorf AG, Hamburg, Germany). The supernatant was removed and separated by 15% SDS-PAGE, and transferred onto a polyvinylidene difluoride membrane (Millipore, Bedford, MA). Membrane blots were probed with mouse anti-Tau (TAU-5) monoclonal antibody (Santa Cruz Biotechnology, Santa Cruz, CA) and visualized by enhanced chemiluminescent.

### Western blot

The separated proteins were transblotted onto a polyvinylidene difluoride membrane at 200 mA for 1 h. The membranes were then blocked with Tris-buffered saline containing 0.05% Tween-20 and 5% dry milk, and incubated sequentially with primary and secondary antibodies dissolved in the same solution for 1 h at room temperature. Endogenous human Tau was recognized by TAU-5 firstly and overexpressed human PDI was recognized by anti-HA antibody secondly. Mouse monoclonal anti-HA antibody (Sigma-Aldrich) was used at 1∶5,000 dilution, TAU-5 (Santa Cruz) was used at 1∶200 dilution, and horseradish peroxidase-conjugated goat anti-mouse IgG (Beyotime) was used at 1∶1,000 dilution. The protein recognized by the antibody was visualized using BeyoECL Plus kit (Beyotime), according to the manufacturer's instructions.

### Immunofluorescence microscopy

For staining, SH-SY5Y cells were fixed with 4% paraformaldehyde for 30 min at room temperature, followed by incubation with 0.25% Triton X-100 for 5 min and blocked with 5% bovine serum albumin for 30 min at room temperature. The cells were then incubated with primary antibody, rabbit polyclonal anti-HA antibody (Proteintech Group, Chicago, IL) or TAU-5 (Santa Cruz), at 37°C for 2 h, followed by incubation with fluorescent secondary antibody, Alexa Fluor 488 or Alexa Fluor 350 (Beyotime), at 37°C for 1 h. Finally, the ER of the cells was stained with ER-Tracker Red (Beyotime) at 37°C for 30 min. Images were captured using an Olympus FluoView FV1000 laser scanning confocal microscope (Tokyo, Japan). Green fluorescence was detected using the 488-nm laser line of an Argon laser, blue fluorescence was detected using the 405-nm laser line of a 405-30 Diode laser, and red fluorescence was detected using the 559-nm laser line of the HeNe laser.

### Thioflavin T binding assays

A 2.5 mM ThT stock solution was freshly prepared in 10 mM HEPES buffer (pH 7.4) and passed through a 0.22- µm pore size filter before use to remove insoluble particles. Under standard conditions, 10 µM Tau_244-372_ was incubated without agitation in 10 mM HEPES buffer (pH 7.4) containing 1 mM DTT and 20 µM ThT with or without human PDI at 37°C for up to 40 min in the presence of fibrillization inducer heparin used in a Tau : heparin molar ratio of 4∶1. The fluorescence of ThT was excited at 440 nm with a slit-width of 7.5 nm and the emission was measured at 480 nm with a slit-width of 7.5 nm on an LS-55 luminescence spectrometer (PerkinElmer Life Sciences, Shelton, CT).

The polymerization for Tau_244-372_ in 96-well plates was set up by a mixture of 10 µM Tau protein, 2.5 µM heparin, 20 µM ThT and 0-20 µM human PDI either in the absence or in the presence of 100 g/l Ficoll 70 in 10 mM HEPES buffer containing 1 mM DTT and 100 mM NaCl (pH 7.4). The reaction components were mixed quickly and immediately read for 10 h at 37°C in SpectraMax M2 microplate reader (Molecular Devices, Sunnyvale, CA) using excitation at 440 nm and emission at 480 nm with a wavelength cut off at 475 nm. Each sample was run in triplicate. Kinetic parameters were determined by fitting ThT fluorescence intensity *versus* time to a sigmoidal equation [4], [38]–[40]:
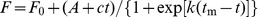
(1)where *F* is the fluorescence intensity, *k* is the rate constant for the growth of fibrils, and *t*_m_ is the time to 50% of maximal fluorescence. The initial baseline during the lag time is described by *F*_0_. The final baseline after the growth phase has ended is described by *A*+*ct*. The lag time is calculated as *t*_m_ − 2/*k*.

### Seeding experiments

Mature fibrils formed by Tau_244–372_ were sonicated on ice using a probe sonicator for 15 s (interval of 3 s, 200 W) to produce the seeds used in the seeding experiments. The kinetics of fibril formation of samples with 0.5% (v/v) preformed seed fibrils were mixed quickly and immediately read for 10 h at 37°C in SpectraMax M2 microplate reader as described above. Each sample was run in triplicate.

### Sarkosyl-insoluble Tau SDS-PAGE

The Sarkosyl-insoluble Tau experiments were carried out according to the method as described [4]. Tau polymerization was set up by incubating a mixture of 10 µM Tau_244–372_, 2.5 µM heparin, and 0–20 µM human PDI in 10 mM HEPES buffer containing 1 mM DTT and 100 mM NaCl (pH 7.4) at 37°C without agitation. Aliquots (100 µl) of assembly mixtures were taken out and added into 500 µl of 10 mM HEPES buffer (pH 7.4) containing 100 mM NaCl and 1% Sarkosyl. The mixture was left at room temperature for 30 min and then centrifugated on an Optima LE-80K ultracentrifuge (Beckman Coulter, Fullerton, CA) at 150,000 *g* for 30 min. The supernatant (sarkosyl-soluble Tau) was removed, and the pellet (sarkosyl-insoluble Tau) was re-suspended in 50 µl of SDS sample buffer containing 5% 2-mercaptoethanol and subjected to 15% SDS-PAGE. After the electrophoresis the gels were stained with silver.

### Transmission electron microscopy

The formation of filaments by human Tau fragment was confirmed by electron microscopy of negatively stained samples. 10 µM Tau_244–372_ was incubated with 0–20 µM human PDI in 10 mM HEPES buffer (pH 7.4) containing 100 mM NaCl, 2.5 µM heparin, and 1 mM DTT at 37°C for 600 or 240 min. Sample aliquots of 10 µl were placed on carbon-coated copper grids, and left at room temperature for 1–2 min, rinsed with H_2_O twice, and then stained with 2% (w/v) uranyl acetate for another 1–2 min. The stained samples were examined using an H-8100 (or an H-7000 FA) transmission electron microscope (Hitachi, Tokyo, Japan) operating at 100 kV or an FEI Tecnai G2 20 transmission electron microscope (Hillsboro, OR) operating at 200 kV.

### Isothermal titration calorimetry

ITC experiments on the interaction of full-length human PDI and its fragments with human Tau fragment Tau_244–372_ (or full-length human Tau) in the absence of DTT were carried out at 25.0 and 37.0°C using a VP-ITC titration calorimetry (MicroCal, Northampton, MA). Freshly purified human PDI and freshly purified human Tau were dialyzed against 10 mM HEPES buffer (pH 7.4) containing 100 mM NaCl for three times at 4°C. A solution of 8.1 µM PDI (or 3.24 µM PDI) was loaded into the sample cell (1.43 ml), and a solution of 200 µM Tau_244–372_ (or 40 µM full-length human Tau) was placed in the injection syringe (300 µl). The first injection (5 µl) of 200 µM Tau_244–372_ (or 40 µM full-length human Tau) was followed by 29 injections of 10 µl (or 19 injections of 15 µl). Dilution heats of human Tau were measured by injecting human Tau solution into buffer alone and were subtracted from the experimental curves prior to data analysis. The stirring rate was 300 rpm. The resulting data were fitted to a single set of identical sites model using MicroCal ORIGIN software supplied with the instrument, and the standard molar enthalpy change for the binding,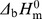, the dissociation constant, *K*_d_, and the binding stoichiometry, *n*, were thus obtained. The standard molar free energy change,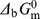, and the standard molar entropy change, 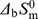, for the binding reaction were calculated by the fundamental equations of thermodynamics [4], [12], [13]:
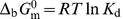
(2)

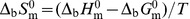
(3)


All ITC experiments were repeated three times. The experiments were pretty reproducible. Every time human Tau possessed a single set of identical sites for human PDI, although the thermodynamic parameters (*K*_d_, 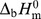 and *n*) were slightly different in different batches.

## Results

### Full-length human PDI Interacted with some endogenous human Tau in vitro

**Figure 1:**
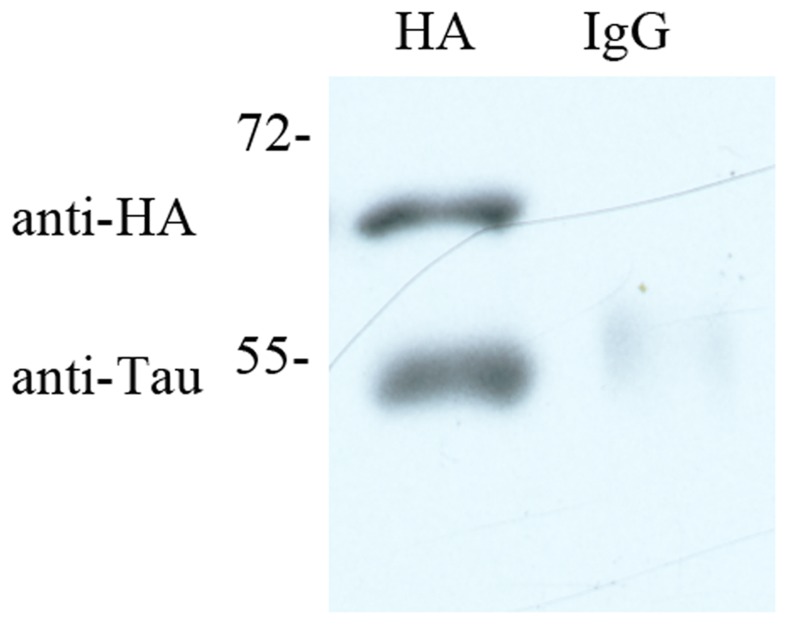
Full-length human PDI interacted with some endogenous human Tau in vitro. SH-SY5Y neuroblastoma cells were transiently transfected with plasmid encoding HA-tagged full-length human PDI. Overexpressed human PDI was immunoprecipitated from cell lysates, and endogenous human Tau was detected by immunoblotting with TAU-5 (left lower panel). Human PDI was also analyzed by Western blot with anti-HA antibody (left upper panel). By contrast, endogenous human Tau was not detected when non-specific IgG antibody was used in immunoprecipitation (right panel).

In this study, we confirmed the interaction of PDI with endogenous Tau in SH-SY5Y cells by co-immunoprecipitation analysis. As shown in Fig. 1, anti-HA antibody was able to pull down endogenous human Tau, which was detected by immunoblotting with TAU-5, and overexpressed human PDI was also detected by Western blot with anti-HA antibody. By contrast, co-immunoprecipitated endogenous human Tau was not detected when non-specific IgG antibody was used in immunoprecipitation (Fig. 1). HA-tagged full-length human PDI co-immunoprecipated with endogenous human Tau, indicating that human PDI and human Tau interacted in SH-SY5Y cells.

**Figure 2:**
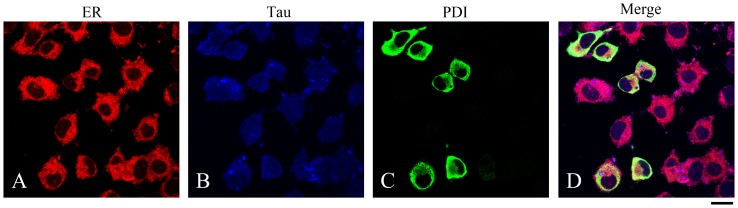
Co-localization of full-length human PDI with endogenous human Tau on the endoplasmic reticulum of SH-SY5Y cells. Confocal microscopy images of immunofluorescence staining for endogenous human Tau (blue) and overexpressed human PDI (green) in SH-SY5Y cells with TAU-5 (B) and anti-HA antibody (C), respectively. Endoplasmic reticulum (ER) (red) was stained with ER-Tracker Red (A). Double and triple labeling of SH-SY5Y cells (Merge) resulted in a strong magenta signal and a strong light cyan signal, respectively (D). The scale bars represent 10 µm.

PDI has been reported to be localized specifically in the lumen of the ER [22]. At first, we performed control experiments to observe the localization of endogenous human Tau in SH-SY5Y cells by using double immunofluorescence. Confocal microscopy image of endogenous human Tau in SH-SY5Y cells in the absence of overexpressed human PDI is shown in Figure S1. Clearly, endogenous human Tau was mainly localized in the nucleus of undifferentiated SH-SY5Y cells and the remaining endogenous human Tau was localized in the cytosol (Fig. S1B), which is similar to the previous observations [41], [42]. Superimposing the two colors blue and red (Merge) resulted in a magenta signal with a blue core (Fig. S1D), indicating that endogenous human Tau was localized both in the nucleus and on the ER of undifferentiated SH-SY5Y cells in the absence of overexpressed human PDI. Immunocytochemical assays were then used to observe the subcellular locations of overexpressed PDI as well as its co-location with cellular endogenous Tau in SH-SY5Y cells. Fig. 2 shows confocal microscopy images of immunofluorescence staining for endogenous human Tau (blue) and human PDI (green) in SH-SY5Y cells with TAU-5 and anti-HA antibody, respectively, as well as fluorescence staining for the ER (red) of the cells with ER-Tracker Red. Superimposing the two colors blue and red (Merge) resulted in a strong magenta signal with a blue core (Fig. 2D), indicating that endogenous human Tau was localized both in the nucleus and on the ER of undifferentiated SH-SY5Y cells when human PDI was overexpressed. Superimposing the three colors green, blue, and red (Merge) resulted in a strong light cyan signal (Fig. 2D), indicating co-localization of full-length human PDI with some endogenous human Tau on the ER of undifferentiated SH-SY5Y cells. Therefore, as evidenced by co-immunoprecipitation and confocal laser scanning microscopy (Figs. 1, 2, and S1), human PDI interacted and co-located with some endogenous human Tau on the ER of undifferentiated SH-SY5Y cells.

### Thermodynamics of the binding of human PDI to human Tau

**Figure 3:**
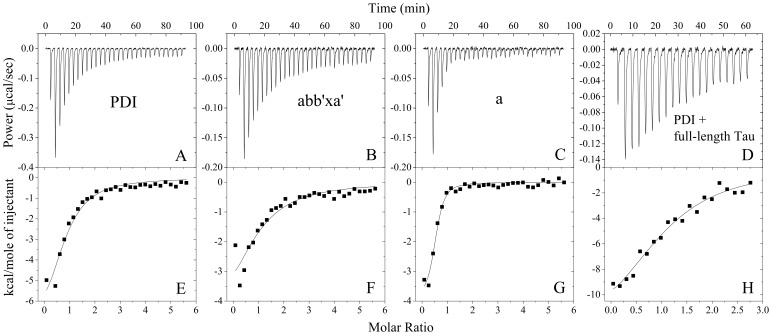
ITC profiles for the binding of full-length human PDI and its fragments to human Tau at 25.0°C. The top panels represent the raw data for sequential injections of 200 µM Tau_244–372_ (or 40 µM full-length human Tau) into 8.1 µM full-length human PDI (A) and its fragments abb’xa’ (B) and a (C) (or 3.24 µM full-length human PDI, D) in 10 mM HEPES buffer (pH 7.4) containing 100 mM NaCl, respectively. The first injection (5 µl) was followed by 29 injections of 10 µl for A-C and 19 injections of 15 µl for D, respectively. The bottom panels (E, F, G, and H) show the plots of the heat evolved (kcal) per mole of human Tau added, corrected for the heat of human Tau dilution, against the molar ratio of human Tau to human PDI. The data (solid squares) were best fitted to a single set of identical sites model and the solid lines represent the best fit.

ITC provides a direct route to the complete thermodynamic characterization of non-covalent, equilibrium interactions [4], [12], [13], [43], and DTT concentrations as low as 1 mM can cause severe baseline artifacts due to background oxidation during the titration. Therefore ITC was used to measure the binding affinity of human PDI to human Tau monomer in the absence of DTT. ITC profiles for the binding of full-length human PDI and its fragments to human Tau fragment Tau_244–372_ at 25.0 and 37.0°C are shown in Figs. 3 and S3, respectively. The top panels in Fig. 3 representatively show raw ITC curves resulting from the injections of Tau_244–372_ into a solution of full-length human PDI (Fig. 3A), domain combination abb’xa’ (Fig. 3B), and domain a (Fig. 3C) at 25.0°C, and the top panels in Fig. S3 representatively show those of Tau_244–372_ titration into a solution of full-length human PDI (Fig. S3A) and domain a (Fig. S3B) at 37.0°C. The titration curves show that PDI binding to Tau_244–372_ is exothermic, resulting in negative peaks in the plots of power versus time. The bottom panels in Fig. 3 show the plots of the heat evolved per mole of Tau_244–372_ added, corrected for the heat of Tau_244–372_ dilution, against the molar ratio of Tau_244–372_ to full-length human PDI (Fig. 3E for 25.0°C and Fig. S3C for 37.0°C), domain combination abb’xa’ (Fig. 3F), and domain a (Fig. 3G for 25.0°C and Fig. S3D for 37.0°C). The calorimetric data were best fit to a model assuming a single set of identical sites. The thermodynamic parameters for the binding of full-length human PDI and its fragments to Tau_244–372_ are summarized in Table 1. As shown in Table 1, at physiological pH, one full-length human PDI bound to one Tau_244–372_ molecule with a dissociation constant of 3.46 µM at 25.0°C and 4.83 µM at 37.0°C. The binding affinity of the four thioredoxin domains abb’xa’ of human PDI to Tau_244–372_ monomer was moderately lower than that of full-length human PDI, with a dissociation constant of 7.35 µM, but the binding affinity of the a-domain of human PDI to Tau_244–372_ monomer was significantly higher than that of full-length human PDI, with a dissociation constant of 0.48 µM at 25.0°C and 1.34 µM at 37.0°C (Table 1). No binding reaction for domain combinations bb’x and a’c with Tau_244–372_ monomer was detected by ITC (Fig. S2A-2D and Table 1), demonstrating that the a-domain of human PDI is very important for the interaction of human PDI with human Tau at physiological pH.

**Table 1:** Thermodynamic parameters for the binding of full-length human PDI and its fragments to human Tau fragment Tau_244–372_ (or full-length human Tau) as determined by ITC at 25.0°C.

PDI	*K*_d_ ( µM)	*n*	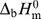 (kcal mol^−1^)	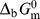 (kcal mol^−1^)	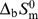 (cal mol^−1^ K^−1^)
Full-length PDI	3.46±0.89	0.80±0.10	−8.60±1.33	−7.45±0.15	−3.84±4.96
abb’xa’	7.35±3.34	0.97±0.32	−5.90±2.40	−7.00±0.27	3.72±8.96
bb’x	NB	−	−	−	−
a’c	NB	−	−	−	−
a	0.48±0.11	0.489±0.020	−4.00±0.20	−8.62±0.14	15.5±1.1
Full-length PDI	4.83±1.92^a^	1.02±0.20^a^	−9.27±2.32^a^	−7.54±0.25^a^	−5.57±8.27^a^
a	1.34±0.41^a^	0.560±0.053^a^	−4.34±0.51^a^	−8.34±0.19^a^	12.9±2.3^a^
Full-length PDI	1.81±0.48^b^	1.12±0.10^b^	−14.5±1.9^b^	−7.83±0.16^b^	−22.2±6.9^b^

Thermodynamic parameters, *K*_d_, 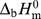 and *n*, were determined using a single set of identical sites model. The standard molar binding free energy (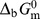) and the standard molar binding entropy (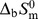) for the binding reaction were calculated using Equations 2 and 3 respectively. The buffer used was 10 mM HEPES buffer (pH 7.4) containing 100 mM NaCl. Errors shown are standard errors of the mean.

NB, no binding observed in the present conditions.

a Thermodynamic parameters for the binding of full-length human PDI and its a-domain to Tau_244–372_ as determined by ITC at 37.0°C.

b Thermodynamic parameters for the binding of full-length human PDI to full-length human Tau as determined by ITC at 25.0°C.

So far, our ITC experiments were conducted with human Tau fragment Tau_244–372_, but since endogenous human Tau is full-length human Tau it is important to demonstrate the thermodynamic behavior of full-length human Tau bound by full-length human PDI. Tau_244–372_ encompasses the four microtubule binding repeats [4], [12]–[16], which does exclude a number of domains that could potentially interact with PDI. ITC profiles for the binding of full-length human PDI to full-length human Tau at 25.0°C are also shown in Fig. 3. Fig. 3D shows raw ITC curves resulting from the injections of full-length human Tau into a solution of full-length human PDI. The titration curves show that full-length human PDI binding to full-length human Tau is also exothermic, resulting in negative peaks in the plots of power versus time. Fig. 3H shows the plots of the heat evolved per mole of full-length human Tau added, corrected for the heat of full-length human Tau dilution, against the molar ratio of human Tau to human PDI. The thermodynamic parameters for the interaction between full-length human PDI and full-length human Tau (Table 1) were obtained by fitting the data to a single set of identical sites model, indicating that one full-length human PDI bound to one full-length human Tau molecule with a moderate affinity in the absence of DTT. The binding affinity of full-length human PDI to full-length human Tau resembles that of full-length human PDI to Tau_244–372_ (Table 1), suggesting that full-length human PDI binds to full-length human Tau mainly through the segment 244–372 of human Tau at physiological pH.

As shown in Table 1, the formation of full-length human PDI-Tau complex was driven by moderately favorable enthalpy decreases but with a large (or moderately) unfavorable entropy decrease for full-length human Tau (or Tau_244–372_). However, the formation of PDI domain combination abb’xa’-Tau complex was driven by moderately favorable increases in entropy in combination with moderately favorable enthalpy decreases, and the formation of the a-domain of human PDI-Tau complex was driven by large favorable increases in entropy in combination with moderately favorable enthalpy decreases (Table 1). Our data indicate that deletion of the entire acidic C-terminal domain of human PDI has significant effect on the “driving force” for the formation of human PDI-Tau complex at physiological pH, although this flexible C-terminal tail has been determined to be not essential for chaperone and enzymatic activities of human PDI [44].

Taken together, our ITC data demonstrated that one full-length human PDI bound to one full-length human Tau (or human Tau fragment Tau_244–372_) monomer with moderate, micromolar affinity at physiological pH and near physiological ionic strength, and that two molecules of the a-domain of human PDI interacted with one Tau_244–372_ molecule with sub-micromolar affinity. Our data also indicated that at physiological pH, human PDI bound to Tau protein mainly through its thioredoxin-like catalytic domain a, forming a 1∶1 complex.

### The presence of human PDI influenced Tau aggregation

**Figure 4:**
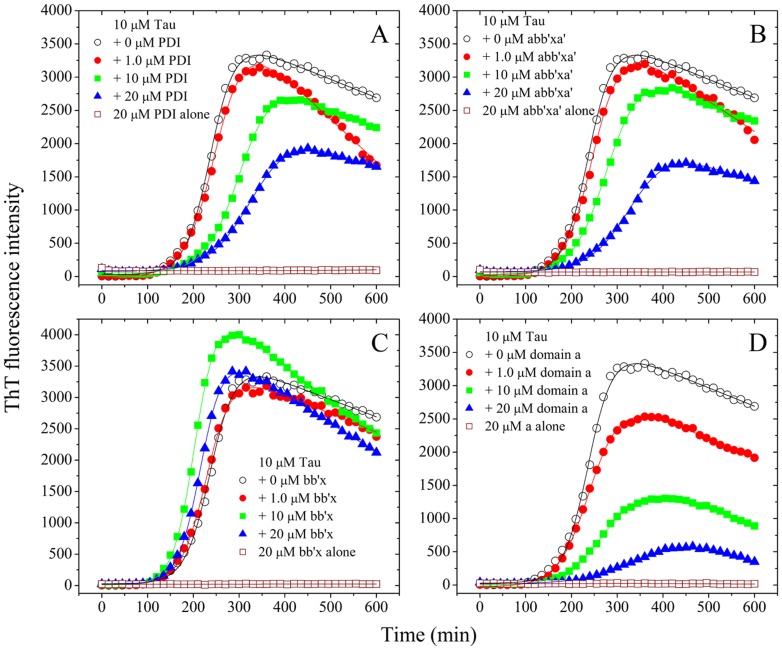
The presence of human PDI altered Tau_244–372_ fibrillization kinetics. 10 µM human Tau fragment Tau_244–372_ was incubated with 0–20 µM full-length human PDI (A) and its fragments abb’xa’ (B), bb’x (C), and a (D) (open circle-0 µM PDI, solid circle-1.0 µM PDI, solid square-10 µM PDI, solid triangle-20 µM PDI, and open square-20 µM PDI alone as control). A sigmoidal equation was fitted to the data and the solid lines represented the best fit. The buffer used was 10 mM HEPES buffer (pH 7.4) containing 100 mM NaCl, 2.5 µM heparin, 1 mM DTT, and 20 µM ThT, and ThT binding assays were carried out at 37°C.

The enhanced fluorescence emission of the dye ThT has been frequently used for monitoring the kinetics of amyloid fibril formation of Tau protein and prion protein, which is a specific marker for the β-sheet conformation of fibril structures [4], [13]–[15], [39], [40]. Neuronal cells normally have a reducing environment maintained by an excess of glutathione [1], [4], [11], [12]. In this study, DTT, a strong reducing agent, was used to mimic the reducing environment present in normal neuronal cells and block the formation of an intramolecular disulfide bond. In this study, human PDI at physiological concentrations was incubated separately with physiological concentrations of recombinant human Tau fragment Tau_244–372_ at physiological pH and in the presence of 1 mM DTT, and the effects of full-length human PDI and its fragments on kinetics of Tau_244–372_ fibrillization were examined by ThT binding assays (Fig. 4), as a function of PDI concentration. As shown in Fig. 4, A and B, the addition of 10–20 µM full-length human PDI or domain combination abb’xa’ did inhibit Tau_244–372_ fibrillization at physiological pH, accompanied by a remarkable decline of the maximum ThT intensity. When the concentration of PDI went higher, the kinetic curves of Tau_244–372_ fibrillization went down gradually, with a longer lag phase.

To elucidate the mechanisms of the inhibitory effect of human PDI on Tau_244–372_ fibrillization, a sigmoidal equation [4], [38]–[40] was used to fit the kinetic data, yielding three kinetic parameters *k*, *t*_m_, and the lag time, which are summarized in Table 2. As shown in Table 2, full-length human PDI and its fragments abb’xa’ remarkably inhibited both steps of nucleation and elongation of Tau_244–372_ fibrillization in a concentration-dependent manner, resulting in a lag time of 242 min (or 247 min) in the presence of 20 µM full-length human PDI (or 20 µM abb’xa’), which is 30% increased compared to that in the absence of PDI (184 min), and a rate constant for the growth of fibrils of 22.4×10^−3^ min^−1^ (or 23.3×10^−3^ min^−1^), which is 40% decreased compared to that in the absence of PDI (36.7×10^−3^ min^−1^). The addition of 20 µM full-length human PDI or domain combination abb’xa’ significantly decreased the amount of Tau_244–372_ filaments represented by the maximum ThT intensity (Fig. 4, A and B). Furthermore, we found that the a-domain of human PDI inhibited both steps of nucleation and elongation of Tau_244–372_ fibrillization more strongly than full-length human PDI (Table 2 and Fig. 4D), resulting in a lag time of 253 min in the presence of 20 µM domain a, which is 40% increased compared to that in the absence of PDI, and a rate constant for the growth of fibrils of 17.6×10^−3^ min^−1^, which is 52% decreased compared to that in the absence of PDI. The addition of 20 µM domain a strongly decreased the amount of Tau_244–372_ filaments represented by the maximum ThT intensity (Fig. 4D), but domain combinations bb’x and a’c of human PDI had no obvious inhibitory effect on the amount of fibrils and fibrillization kinetics of Tau_244–372_ in reducing conditions (Table 2 and Fig. 4C), demonstrating that the a-domain of human PDI is very important for the inhibitory effect of human PDI on Tau fibrillization at physiological pH. Our data indicated that human PDI prevented abnormal Tau aggregation mainly through its thioredoxin-like catalytic domain a.

**Table 2:** Kinetic parameters of Tau_244–372_ fibrillization in the presence of different concentrations of full-length human PDI and its fragments as determined by ThT binding assays at 37°C.

PDI	[PDI] ( µM)	*k* (10^−3^ min^−1^)	*t*_m_ (min)	Lag time (min)
Full-length PDI	0	36.7±1.1	238.3±1.1	184±3
	1.0	30.9±0.8	251.7±1.0	187±3
	10	27.6±0.7	303.4±1.3	231±3
	20	22.4±0.5	331.4±1.9	242±4
	0	54.9±3.7^a^	11.6±3.8^a^	∼0^a^
	20	31.4±1.0^a^	108.5±1.2^a^	44.8±3^a^
abb’xa’	0	36.7±1.1	238.3±1.1	184±3
	1.0	32.0±0.9	249.7±1.1	187±3
	10	27.4±0.5	280.4±1.0	207±2
	20	23.3±0.9	333.2±2.9	247±6
bb’x	0	36.7±1.1	238.3±1.1	184±3
	1.0	35.7±1.0	230.4±1.0	174±3
	10	43.0±0.6	204.2±0.4	158±1
	20	40.5±0.6	217.8±0.4	168±1
a’c	0	36.7±1.1	238.3±1.1	184±3
	1.0	34.9±0.8	231.5±0.8	174±2
	10	31.7±1.0	251.6±1.3	188±3
	20	24.6±0.3	301.9±0.8	220±2
a	0	36.7±1.1	238.3±1.1	184±3
	1.0	29.5±0.6	241.8±0.9	174±2
	10	23.9±0.6	278.2±1.6	195±4
	20	17.6±0.4	366.3±3.3	253±6

Kinetic parameters, *k*, *t*_m_, and the lag time, were determined by fitting ThT fluorescence intensity *versus* time to Equation 1. The final concentration of human Tau fragment Tau_244–372_ was 10 µM. The buffer used was 10 mM HEPES buffer (pH 7.4) containing 1 mM DTT, 100 mM NaCl, 2.5 µM heparin and 20 µM ThT. Errors shown are standard errors of the mean.

a Kinetic parameters of Tau_244–372_ fibrillization in the presence of 100 g/l Ficoll 70 and different concentrations of full-length human PDI as determined by ThT binding assays at 37°C.

∼, Observed from the ThT fluorescence curves directly.

In order to check whether human PDI has an inhibitory effect on Tau fibrillization in the presence of seeds, we carried out seeding experiments. As shown in Figs. S4 and 4, in the absence of PDI, the addition of seeds significantly reduced the lag time of Tau_244–372_ fibrillization. The addition of 20 µM full-length human PDI remarkably inhibited both steps of nucleation and elongation of seed-induced Tau_244–372_ fibrillization (Fig. S4). Furthermore, we found that the a-domain of human PDI (20 µM) not only inhibited both steps of seed-induced Tau_244–372_ fibrillization more strongly than full-length human PDI, but also significantly decreased the amount of Tau_244–372_ filaments represented by the maximum ThT intensity (Fig. S4). Clearly, human PDI did have an inhibitory effect on Tau fibrillization in the presence of seeds. This is probably due to the binding between Tau seeds and human PDI, so that the amyloid elongation via lateral association of Tau seeds/monomer was blocked.

Ficoll 70 is widely accepted as a perfect model for the principal crowding components in living cells where the folding and misfolding of proteins take place, because its interaction with proteins can be described using pure excluded-volume models [40], [45]. In this study, the effect of such a macromolecular crowding agent on human Tau filament formation suppressed by human PDI under reducing conditions was examined by ThT binding assays (Table 2). As shown in Table 2, the presence of 100 g/l Ficoll 70 in the reaction systems significantly accelerated amyloid fibril formation of Tau_244–372_ in the absence of PDI, almost without a lag phase. However, 20 µM full-length human PDI strongly inhibited both steps of nucleation and elongation of Tau_244–372_ fibrillization in the presence of 100 g/l Ficoll 70, resulting in a lag time of 44.8 min, which is infinite increase compared with that in the absence of PDI (0 min), and a rate constant for the growth of fibrils of 31.4×10^−3^ min^−1^, which is 43% decreased compared to that in the absence of PDI (54.9×10^−3^ min^−1^) (Table 2). The above results indicated that the inhibitory effect of human PDI on the nucleation step of Tau fibrillization under crowded physiological conditions is much stronger than that in dilute solutions.

### The amount of Tau filaments present in the solution measured by centrifugation assays

**Figure 5:**
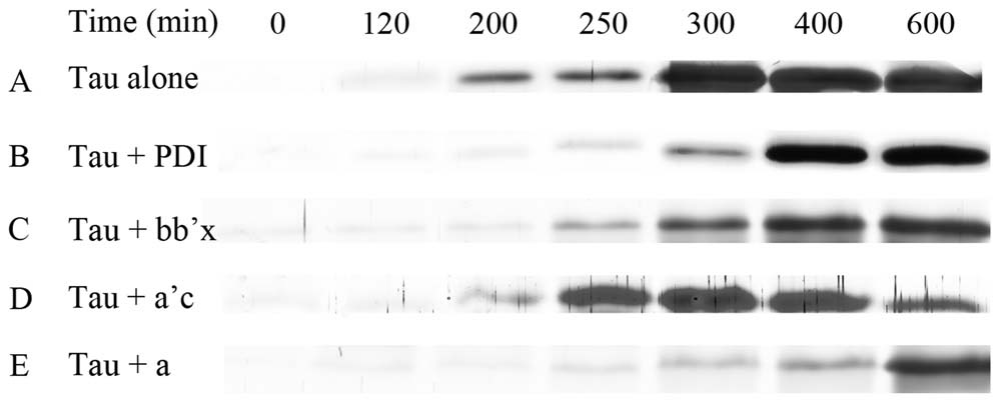
Time-dependent SDS-PAGE analysis of Sarkosyl-insoluble Tau_244–372_ in the presence of human PDI. 10 µM human Tau fragment Tau_244–372_ was incubated with 0 µM full-length human PDI (A), 20 µM full-length human PDI (B), 20 µM bb’x (C), 20 µM a’c (D), and 20 µM a (E), respectively, in 10 mM HEPES buffer (pH 7.4) containing 100 mM NaCl, 2.5 µM heparin, and 1 mM DTT at 37°C. Aliquots were taken at 0, 120, 200, 250, 300, 400, and 600 min, respectively, and then incubated with HEPES buffer containing 1% Sarkosyl followed by centrifuging at 150,000 *g* for 30 min. Pellets were re-suspended with sample buffer containing 5% 2-mercaptoethanol and subjected to 15% SDS-PAGE. Gels were stained with silver.

ThT fluorescence is not perfectly specific for amyloid fibrils and, depending on the particular protein and experimental conditions, assays may render both false positive (spectroscopic change upon binding to non-fibrillar material) and false negative results (its fluorescence not being affected by some amyloid fibrils) [14]. Considering this, we investigated the correlation between the spectroscopic signal monitored and the amount of Tau filaments present in the solution measured by centrifugation assays. In order to semi-quantify the increase/decrease of Tau filaments in the presence of human PDI, we carried out Sarkosyl-insoluble SDS-PAGE experiments after centrifugation assays. As shown in Fig. 5, A, B, and E, a clear band corresponding to Sarkosyl-insoluble Tau_244–372_ filaments was observed when Tau_244–372_ was incubated in the absence of a physiological inhibitor human PDI for 200 min, while the Sarkosyl-insoluble Tau band was clearly observed when Tau_244–372_ was incubated with 20 µM full-length human PDI for a remarkably longer time (300 min) or incubated with 20 µM domain a for a much longer time (400 min). Furthermore, when Tau_244–372_ was incubated for 300/400/600 min, the intensity of the Sarkosyl-insoluble Tau band in the presence of 20 µM full-length human PDI was remarkably lower than that in the absence of PDI, and the intensity of the Sarkosyl-insoluble Tau band in the presence of 20 µM domain a was much lower than that in the absence of PDI (Fig. 5, A, B, and E). All Sarkosyl-insoluble SDS-PAGE experiments were repeated three times in order to get a more quantitative result. Fig. S5 shows the quantitative characterization of time-dependent SDS-PAGE of Sarkosyl-insoluble Tau_244–372_ in the presence of human PDI, compared with that in the absence of PDI. As shown in Fig. S5, A, B, and C, the addition of 20 µM full-length human PDI or 20 µM the a-domain resulted in a lag time of Tau_244–372_ fibrillization remarkably longer than that in the absence of PDI. These findings further support the observations mentioned above that the addition of 20 µM full-length human PDI did inhibit Tau_244–372_ fibrillization at physiological pH, and that the a-domain of human PDI inhibited Tau_244–372_ fibrillization more strongly than full-length human PDI. However, domain combinations bb’x and a’c of human PDI had no obvious inhibitory effect on Tau_244–372_ fibrillization under reducing conditions (Fig. 5, C and D), further demonstrating that the a-domain of human PDI is important for the inhibitory effect of human PDI on Tau fibrillization at physiological pH.

### Characterization of the morphology of Tau protein aggregates formed in the presence of human PDI

**Figure 6:**
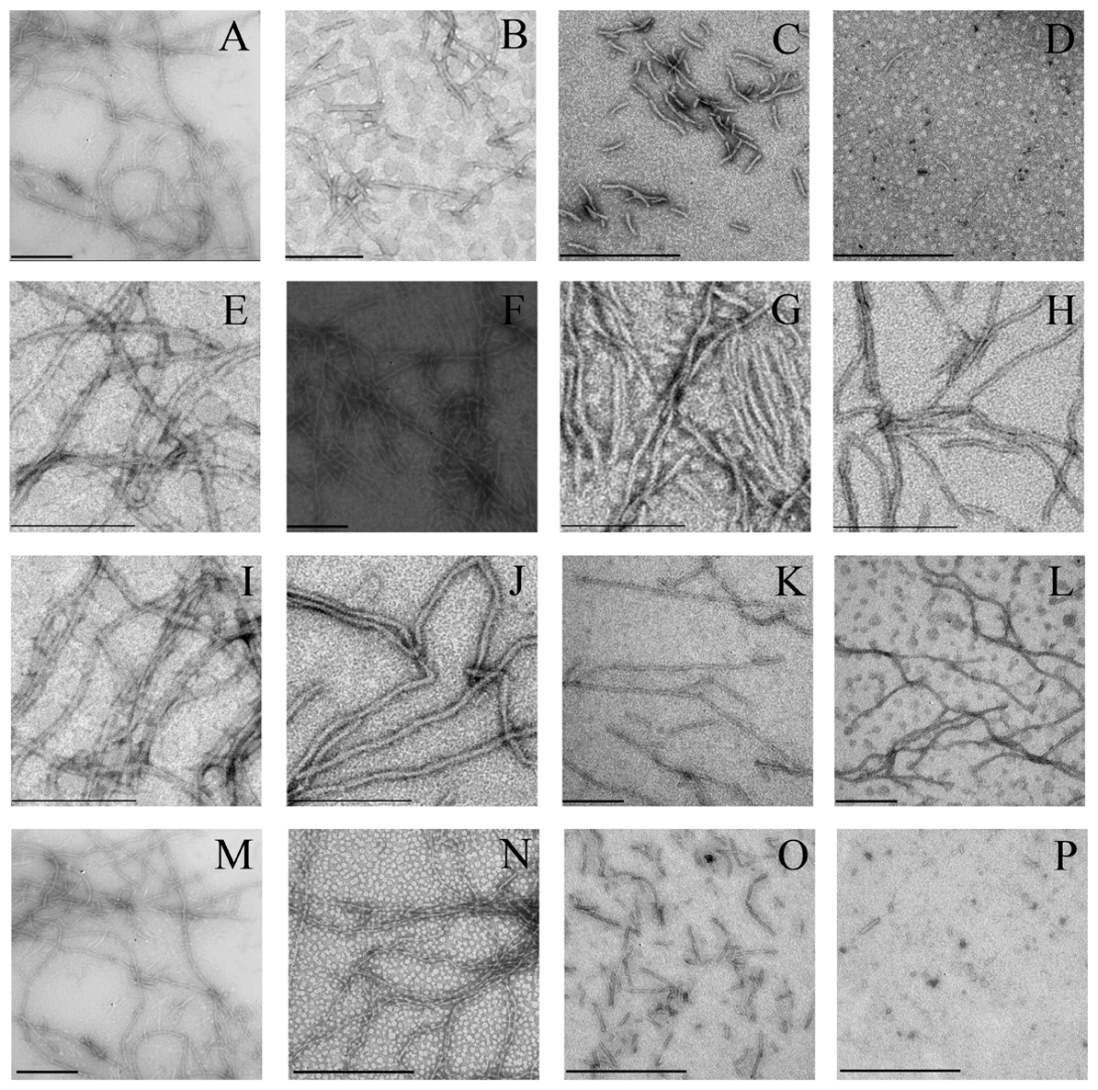
Transmission electron micrographs of Tau_244–372_ aggregates formed with human PDI. 10 µM human Tau fragment Tau_244–372_ was incubated with 0–20 µM full-length human PDI (A-0 µM, B-1.0 µM, C-10 µM, and D-20 µM), 0-20 µM bb’x (E-0 µM, F-1.0 µM, G-10 µM, and H-20 µM), 0–20 µM a’c (I-0 µM, J-1.0 µM, K-10 µM, and L-20 µM), and 0–20 µM a (M-0 µM, N-1.0 µM, O-10 µM, and P-20 µM), respectively, in 10 mM HEPES buffer (pH 7.4) containing 100 mM NaCl, 2.5 µM heparin, and 1 mM DTT at 37°C for 600 min. A 2% (w/v) uranyl acetate solution was used to negatively stain the fibrils. The scale bars represent 200 nm.

**Figure 7:**
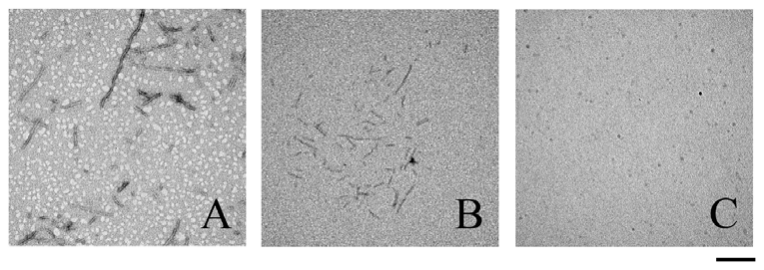
Transmission electron micrographs of Tau_244–372_ samples after incubation under different conditions. 10 µM human Tau fragment Tau_244–372_ was incubated with 0–20 µM full-length human PDI (A-0 µM and B-20 µM) and 0–20 µM a (A-0 µM and C-20 µM), respectively, in 10 mM HEPES buffer (pH 7.4) containing 100 mM NaCl, 2.5 µM heparin, and 1 mM DTT at 37°C for 240 min. A 2% (w/v) uranyl acetate solution was used to negatively stain the fibrils. The scale bars represent 200 nm.

TEM was employed to characterize the morphology of Tau protein aggregates formed in the absence and in the presence of human PDI. Fig. 6 shows TEM images of Tau_244–372_ samples incubated with 20 µM human PDI under reducing conditions and at 37°C for 600 min (the final stage). For Tau_244–372,_ the addition of 1 µM full-length human PDI or 1 µM the a-domain had no significant effect on the morphology of Tau samples, and long and branched fibrils were observed in all samples (Fig. 6, A, B, M, and N). In the presence of 10 µM full-length human PDI or 10 µM the a-domain, however, some short filaments were observed when Tau samples were incubated for 600 min (Fig. 6, C and O). The amount of fibrils formed by Tau_244–372_ in the presence of 10 µM full-length human PDI or 10 µM the a-domain (Fig. 6, C and O) appears to be markedly less than that in the absence of PDI (Fig. 6, A and M) on the same time scale. In the presence of 20 µM full-length human PDI (or 20 µM the a-domain), only a few short amyloid fibrils (or almost no fibrils) were observed when Tau samples were incubated for 600 min (Fig. 6, D and P). As expected, for Tau_244–372,_ the addition of 10–20 µM domain combination bb’x or 10–20 µM a’c had no significant effect on the morphology of Tau samples, and long and branched fibrils were observed in all samples (Fig. 6, E-L). Furthermore, we semi-quantitatively analyzed the inhibitory effect of PDI on Tau fibrillization by using TEM. Fig. 7 shows TEM images of Tau_244–372_ samples incubated with 20 µM human PDI under reducing conditions and at 37°C for 240 min (the early stage). In the absence of PDI, some short amyloid fibrils accompanied by a few long fibrils were observed when Tau samples were incubated for 240 min (Fig. 7A). In the presence of 20 µM full-length human PDI, however, some shorter and thinner protofibrils were observed when Tau samples were incubated for 240 min (Fig. 7B). In the presence of 20 µM the a-domain, no fibrils were observed when Tau samples were incubated for 240 min (Fig. 7C). These findings once again support the observations that the addition of 10–20 µM full-length human PDI did inhibit Tau_244–372_ fibrillization at physiological pH, and that the a-domain of human PDI inhibited Tau_244–372_ fibrillization more strongly than full-length human PDI.

### Characterization of the effect of human PDI on Tau fibrillization at different stages

**Figure 8:**
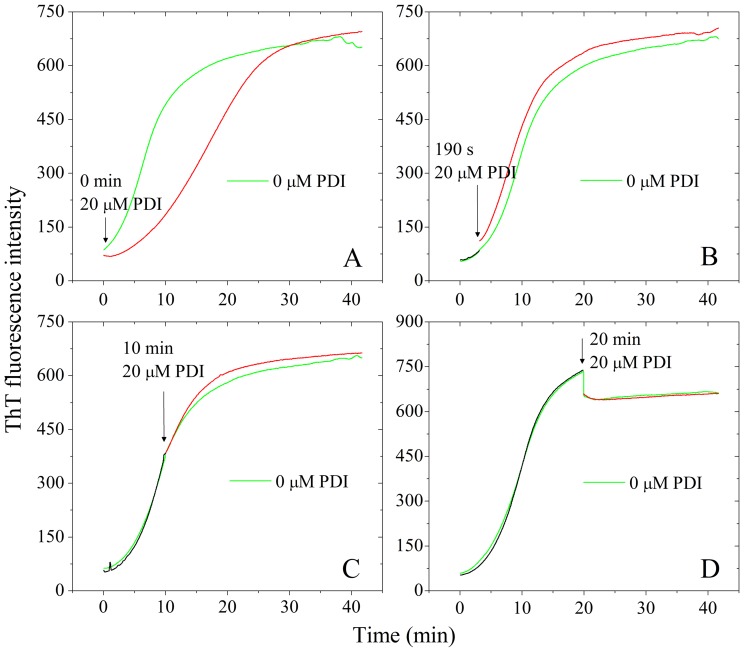
Effect of human PDI on Tau_244–372_ fibrillization at various time points. 10 µM human Tau fragment Tau_244–372_ was incubated without full-length human PDI (black curves) and then titrated with 20 µM full-length human PDI at 0 min (A), 190 s (B), 10 min (C), and 20 min (D) respectively (red curves). The beginning of titrations is indicated by black arrows, and the curves are compared with 10 µM Tau_244–372_ incubated without human PDI (green curves). The buffer used was 10 mM HEPES buffer (pH 7.4) containing 2.5 µM heparin, 1 mM DTT, and 20 µM ThT, and ThT binding assays were carried out at 37°C.

We then checked whether the inhibitory effect of human PDI on Tau fibrillization was dependent on the incubation time or different compositions of Tau protein. We first added 20 µM full-length human PDI at time zero when Tau_244–372_ was pure monomers. As shown in Fig. 8A, full-length human PDI remarkably inhibited both steps of nucleation and elongation of Tau_244–372_ fibrillization under such conditions. We then added 20 µM full-length human PDI after incubation for 190 s, 10 min, and 20 min, when Tau_244–372_ was oligomers/protofibrils, fibrils, and mature fibrils, respectively. As shown in Fig. 8, B-D, full-length human PDI added at 190 s in the lag phase, 10 min in the growth phase, or at 20 min in the final equilibrium phase, did not inhibit fibril formation of Tau_244–372_ under reducing conditions. Particularly, when human PDI was added after incubation of human Tau for 190 s, which there should be some Tau oligomers (seeds) formed in solution, no inhibition effect was observed (Fig. 8B). The above results suggested that the inhibitory effect on fibrillization kinetics of Tau by full-length human PDI was due to its direct interaction with Tau protein. Such an interaction was solidly demonstrated by co-immunoprecipitation (Fig. 1), confocal laser scanning microscopy (Fig. 2), and isothermal titration calorimetry (Figs. 3 and S3). It is this interaction that decelerated the nucleation of Tau aggregation. The reason why PDI reduced Tau seeds fibrillization but not Tau oligomers fibrillization is unknown.

## Discussion

A hallmark of tauopathies including Alzheimer disease is accumulation of misfolded Tau protein within neurons, forming neurofibrillary tangles and leading to cellular dysfunction and cell death [1]–[3]. Amyloid fibrils associated with Alzheimer disease [46]–[50] can be considered biologically relevant failures of cellular quality control mechanisms including molecular chaperones, proteolysis, autophagy, and proteasomes [51], [52]. As an important molecular chaperone induced by ER stress, PDI is found to co-locate with neurofibrillary tangles in Alzheimer disease patient brain tissue [34], [35] and is believed to prevent the neurotoxicity associated with ER stress and protein misfolding [20], [23]. However, the connection between PDI function and Tau protein misfolding during ER stress remains enigmatic. Our Co-IP and confocal laser scanning microscopy data provided direct evidence for the interaction between human PDI and human Tau in neuronal cells. We demonstrated for the first time that human PDI interacted and co-located with some endogenous human Tau on the ER of SH-SY5Y cells. Our data here clearly showed that at physiological pH, one human PDI molecule bound to one human Tau molecule with moderate, micromolar affinity, and thus remarkably inhibited both steps of nucleation and elongation of Tau protein misfolding in a concentration-dependent manner. Therefore, human PDI formed a 1∶1 complex with human Tau protein and prevented abnormal Tau aggregation under physiological conditions.

In this paper, DTT was not used due to its background noise. We then discussed the possible influence of the absence of DTT toward ITC measurement results. We have previously demonstrated that the extended Tau monomers are analyzed in our ITC measurements, which are made in the absence of DTT [13]. It is possible that the binding affinity of human PDI to Tau monomer in the presence of DTT is higher than that of human PDI to Tau monomer in the absence of DTT.

During neurodegenerative disorders and cerebral ischaemia, the accumulation of immature and denatured proteins or misfolded proteins in the ER results in a cellular condition known as ER stress [19], [20], [53], which triggers an adaptive program called the unfolded protein response [23], [54], [55]. An upregulation of PDI during ER stress [56]–[58] associated with multiple neurodegenerative diseases including Alzheimer disease [20], [23], [32], [33], [58], [59] represents an adaptive response to protect neuronal cells. PDI family members have been reported to prevent abnormal aggregation of several proteins/peptides associated with neurodegenerative diseases through a direct interaction between PDI and these amyloidogenic proteins, including prion proteins [60], [61], α-synuclein [62], amyloid β peptides [63], FUS [64], and copper, zinc superoxide dismutase [59]. Our results here indicate that human PDI, a key component of the protein quality control system in the ER [22], [62], can directly interact with human Tau on the ER, and could counteract the toxicity caused by abnormal Tau aggregation in the ER by binding to Tau protein and inhibiting Tau fibrillization. These results, coupled with some previous studies [23], [59]–[64], highlight the potent effectiveness of PDI in preventing abnormal aggregation of amyloidogenic proteins at the various stages of their aggregation pathway. Therefore, PDI family members can reduce general ER stress levels in protein misfolding disorders as a part of the unfolded protein response [23], [54], [55], prevent the misfolding of amyloidogenic proteins through a direct interaction [23], [59]–[64], and prevent neurotoxicity associated with ER stress and protein misfolding [20], [23], [33], [59]–[61].

Both domains a and a’ of human PDI contain an active site CGHC that is responsible for the oxidoreductase activity [27], [28]. Isolated a and a’ domains have been shown to effectively catalyze the introduction of disulfides into protein substrates [65]. It has been reported that domain a’ is very important for human PDI to inhibit α-synuclein fibril formation [62]. Very recently, it has been found that non-native disulfides are formed early in the folding pathway and can trigger misfolding, but the a-domain of human PDI favors native disulfides by catalyzing oxidation at a late stage of folding [66]. Furthermore, the molecular flexibility of the a-domain of PDI is demonstrated to be essential for the enzymatic activity *in vitro* and *in vivo* [67]. In the present study, we found that two molecules of the a-domain of human PDI interacted with one Tau molecule with sub-micromolar affinity, and inhibited both steps of nucleation and elongation of Tau fibrillization more strongly than full-length human PDI. In other words, the a-domain is very important for the inhibitory effect of human PDI on Tau fibrillization under physiological reducing conditions. Our results indicated that human PDI bound to human Tau mainly through its thioredoxin-like catalytic domain a, forming a 1∶1 complex and preventing abnormal Tau aggregation. We had hoped to purify domain a’ to homogeneity by using Ni Sepharose chromatography, but unfortunately we did not succeed. Although no binding reaction for domain combination a’c with Tau monomer was detected by ITC and a’c had no obvious inhibitory effect on Tau fibrillization under physiological reducing conditions, the possibility that domain a’ interacts with human Tau and inhibits Tau fibrillization to some extent cannot be excluded.

Very recently, it has been demonstrated that operational plasticity enables molecular chaperone Hsp104 to disaggregate diverse amyloid fibrils, including those formed by full-length human Tau and Tau_244–372_ [68]. Our data here suggest that human PDI could act as a physiological inhibitor of Tau fibrillization, protecting ER from the harmful effect of misfolded Tau protein. Our findings are highly relevant to the emerging viewpoint that PDI action is intimately involved in the prevention of *in vivo* amyloid fibril formation [23], [59]–[64]. Physiological inhibitors for Tau fibrillization, such as molecular chaperones Hsp70 [6], [7] and PDI (this work), and molecular chaperones disaggregating Tau filaments, such as Hsp104 [68], hold promise for development of novel strategies for treatment and early diagnosis of Alzheimer disease.

In conclusion we have shown that: (i) full-length human PDI Interacted with endogenous human Tau in SH-SY5Y cells; (ii) full-length human PDI co-located with some endogenous human Tau on the ER of SH-SY5Y cells; (iii) at physiological pH, one human PDI bound to one human Tau monomer with moderate, micromolar affinity and mainly through its thioredoxin-like catalytic domain a; (iv) human PDI prevented abnormal Tau aggregation also mainly through its thioredoxin-like catalytic domain a; (v) the inhibitory effect on fibrillization kinetics of human Tau by human PDI was mainly due to its direct interaction with Tau protein. The above findings can be used to develop novel strategies for treatment of Alzheimer disease. This study provides clues to understand the role of PDI in preventing Tau protein misfolding during ER stress, and offers new insights into pathogenesis, placing ER stress onto a generic pathophysiology for Alzheimer disease and holding the promise to help solve Alzheimer disease.

## Supporting Information

Figure S1 **The localization of endogenous human Tau in SH-SY5Y cells detected by double immunofluorescence – control experiments.** Confocal microscopy image of immunofluorescence staining for endogenous human Tau (blue) in SH-SY5Y cells with TAU-5 (B) in the absence of overexpressed human PDI (C). Endoplasmic reticulum (ER) (red) was stained with ER-Tracker Red (A). Double labeling of SH-SY5Y cells (Merge) resulted in a magenta signal (D). The scale bars represent 10 µm.
(DOC)

Figure S2 **ITC profiles for the binding of human PDI fragments to Tau_244–372_ at 25.0°C.** The top panels represent the raw data for sequential injections of 214 µM Tau_244–372_ into 8.1 µM human PDI fragment bb’x (A) and sequential injections of 336 µM Tau_244–372_ into 16.2 µM human PDI fragment a’c (B) in 10 mM HEPES buffer (pH 7.4) containing 100 mM NaCl, respectively. The first injection (5 µl) was followed by 29 injections of 10 µl. The bottom panels (C and D) show the plots of the heat evolved (kcal) per mole of Tau_244–372_ added, corrected for the heat of Tau_244–372_ dilution, against the molar ratio of Tau_244–372_ to human PDI fragment. The data (solid squares) were too small to be fitted, indicating that no binding was observed in the conditions used.
(DOC)

Figure S3 **ITC profiles for the binding of full-length human PDI and its a-domain to Tau_244–372_ at 37.0°C.** The top panels represent the raw data for sequential injections of 200 µM Tau_244–372_ into 8.1 µM full-length human PDI (A) and its fragment a (B) in 10 mM HEPES buffer (pH 7.4) containing 100 mM NaCl, respectively. The first injection (5 µl) was followed by 29 injections of 10 µl. The bottom panels (C and D) show the plots of the heat evolved (kcal) per mole of Tau_244–372_ added, corrected for the heat of Tau_244–372_ dilution, against the molar ratio of Tau_244–372_ to human PDI fragment. The data (solid squares) were best fitted to a single set of identical sites model and the solid lines represent the best fit.
(DOC)

Figure S4 **Effect of human PDI on seed-induced fibril formation of Tau_244_**_–**372**_ **.** 10 µM human Tau fragment Tau_244–372_ was incubated with 0.5% seeds and 0–20 µM full-length human PDI and domain a (open circle-0 µM PDI, solid circle-20 µM full-length PDI, and solid triangle-20 µM domain a). A sigmoidal equation was fitted to the data and the solid lines represented the best fit. The buffer used was 10 mM HEPES buffer (pH 7.4) containing 100 mM NaCl, 2.5 µM heparin, 1 mM DTT, and 20 µM ThT, and ThT binding assays were carried out at 37°C.
(DOC)

Figure S5 Quantitative characterization of time-dependent SDS-PAGE of Sarkosyl-insoluble Tau_244–372_ in the presence of human PDI, compared with that in the absence of human PDI. Grey scanning for protein band corresponding to Sarkosyl-insoluble Tau_244–372_ in the absence of full-length human PDI (A) and in the presence of 20 µM full-length human PDI (B) and 20 µM domain a (C) is shown by using UN-SCAN-IT gel 6.1 soft.
(DOC)
