# Supplementary figures and images for: Protein Disulfide Isomerase Interacts with Tau Protein and Inhibits Its Fibrillization
Source: PLoS One. 2013 Oct 2;8(10):e76657. doi: 10.1371/journal.pone.0076657 (PMC3788760; doi:10.1371/journal.pone.0076657)

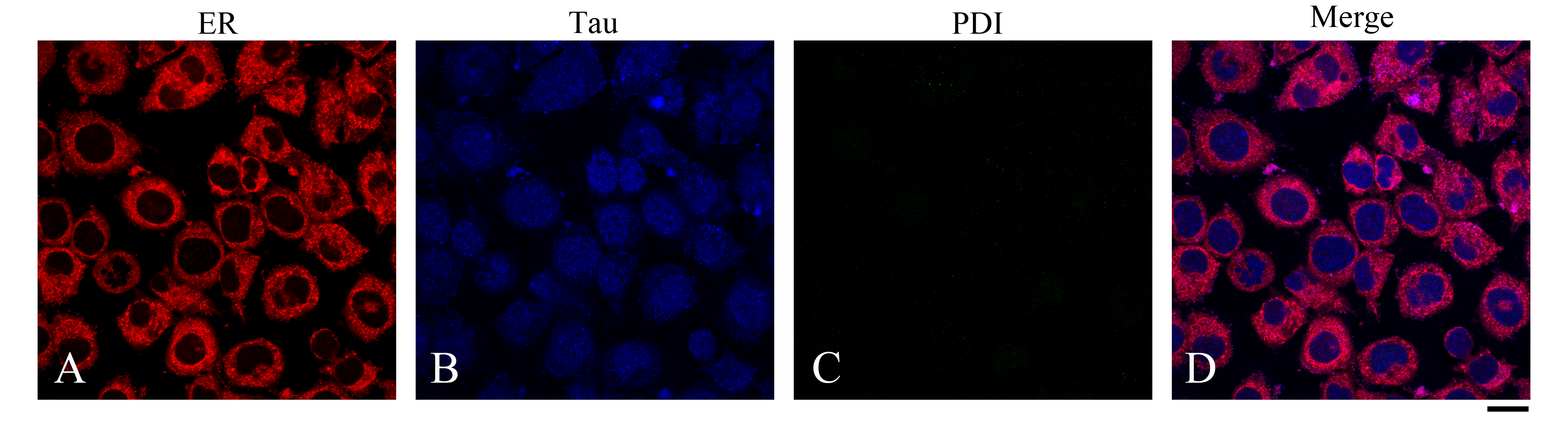

Supplement: Figure S1 — The localization of endogenous human Tau in SH-SY5Y cells detected by double immunofluorescence – control experiments. Confocal microscopy image of immunofluorescence staining for endogenous human Tau (blue) in SH-SY5Y cells with TAU-5 (B) in the absence of overexpressed human PDI (C). Endoplasmic reticulum (ER) (red) was stained with ER-Tracker Red (A). Double labeling of SH-SY5Y cells (Merge) resulted in a magenta signal (D). The scale bars represent 10 µm. (DOC) [file pone.0076657.s001.doc]
