# Supplementary material for: Protein Disulfide Isomerase Interacts with Tau Protein and Inhibits Its Fibrillization
Source: PLoS One. 2013 Oct 2;8(10):e76657. doi: 10.1371/journal.pone.0076657 (PMC3788760; doi:10.1371/journal.pone.0076657)
Supplement: Supplementary file 2 [file pone.0076657.s002.doc]

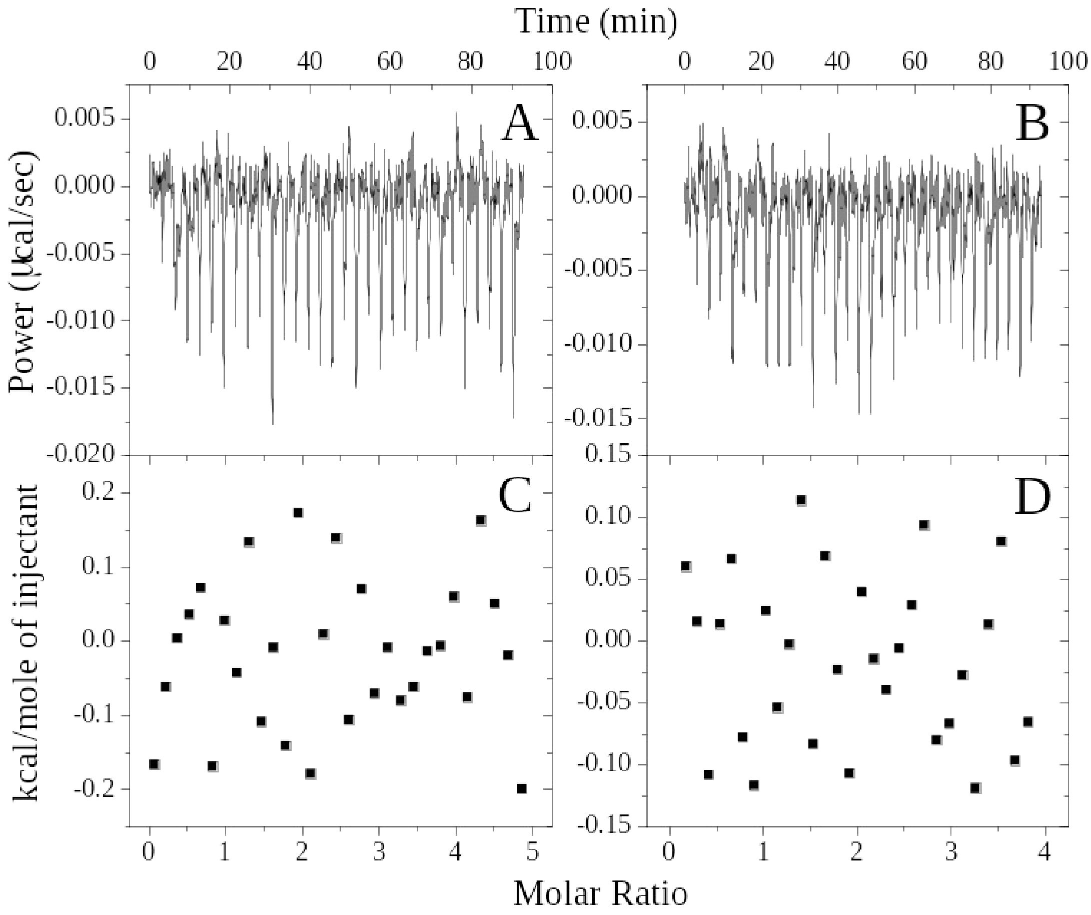
Time (min)
Power (µcal/sec)
kcal/mole of injectant
Molar Ratio
A
B
C
D
