# Supplementary material for: Protein Disulfide Isomerase Interacts with Tau Protein and Inhibits Its Fibrillization
Source: PLoS One. 2013 Oct 2;8(10):e76657. doi: 10.1371/journal.pone.0076657 (PMC3788760; doi:10.1371/journal.pone.0076657)
Supplement: Supplementary file 3 [file pone.0076657.s003.doc]

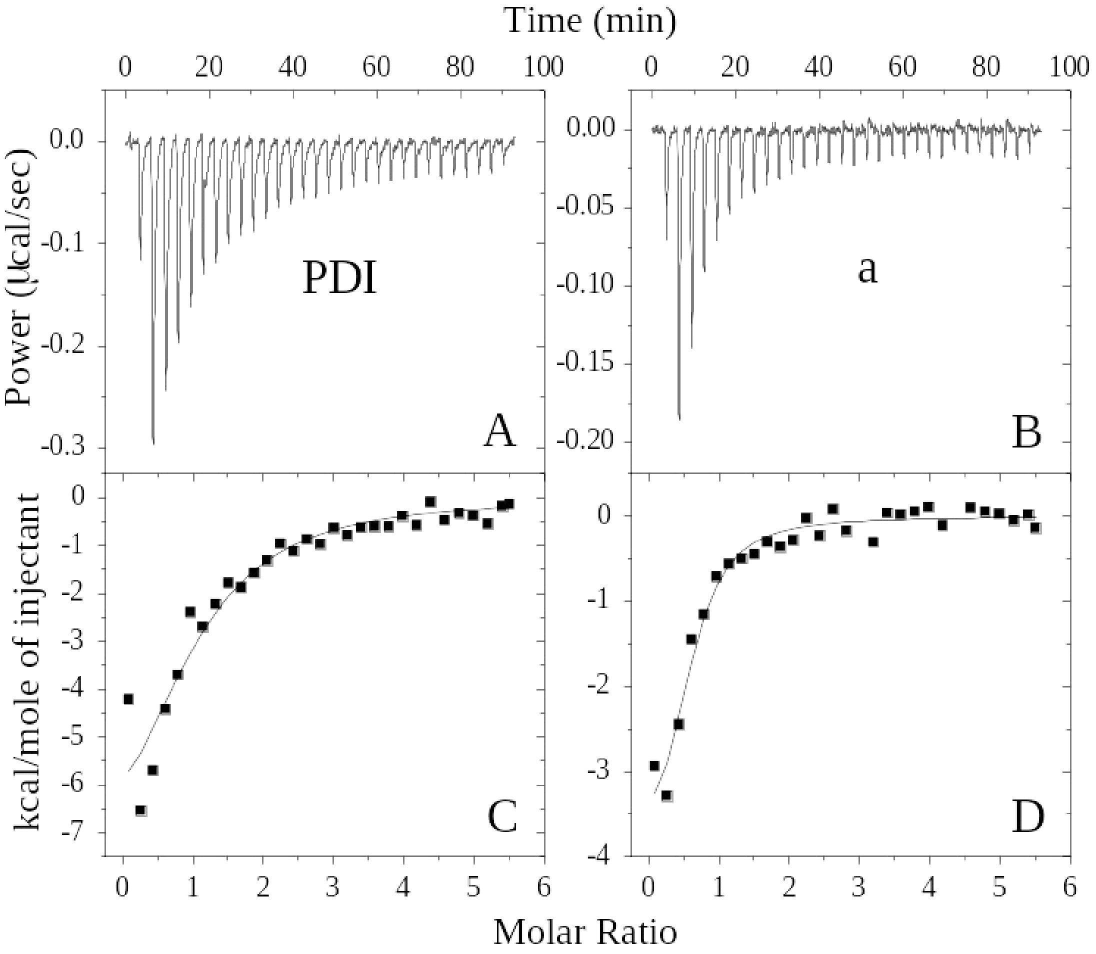

Time (min)
0 20 40 60 80 100
0 20 40 60 80 100
Power (μcal/sec)
0.0
-0.1
-0.2
-0.3
PDI
A
0.00
-0.05
-0.10
-0.15
-0.20
a
B
kcal/mole of injectant
0
-1
-2
-3
-4
-5
-6
-7
C
0
-1
-2
-3
-4
D
0 1 2 3 4 5 6
0 1 2 3 4 5 6
Molar Ratio
