# Supplementary material for: Protein Disulfide Isomerase Interacts with Tau Protein and Inhibits Its Fibrillization
Source: PLoS One. 2013 Oct 2;8(10):e76657. doi: 10.1371/journal.pone.0076657 (PMC3788760; doi:10.1371/journal.pone.0076657)
Supplement: Supplementary file 4 [file pone.0076657.s004.doc]

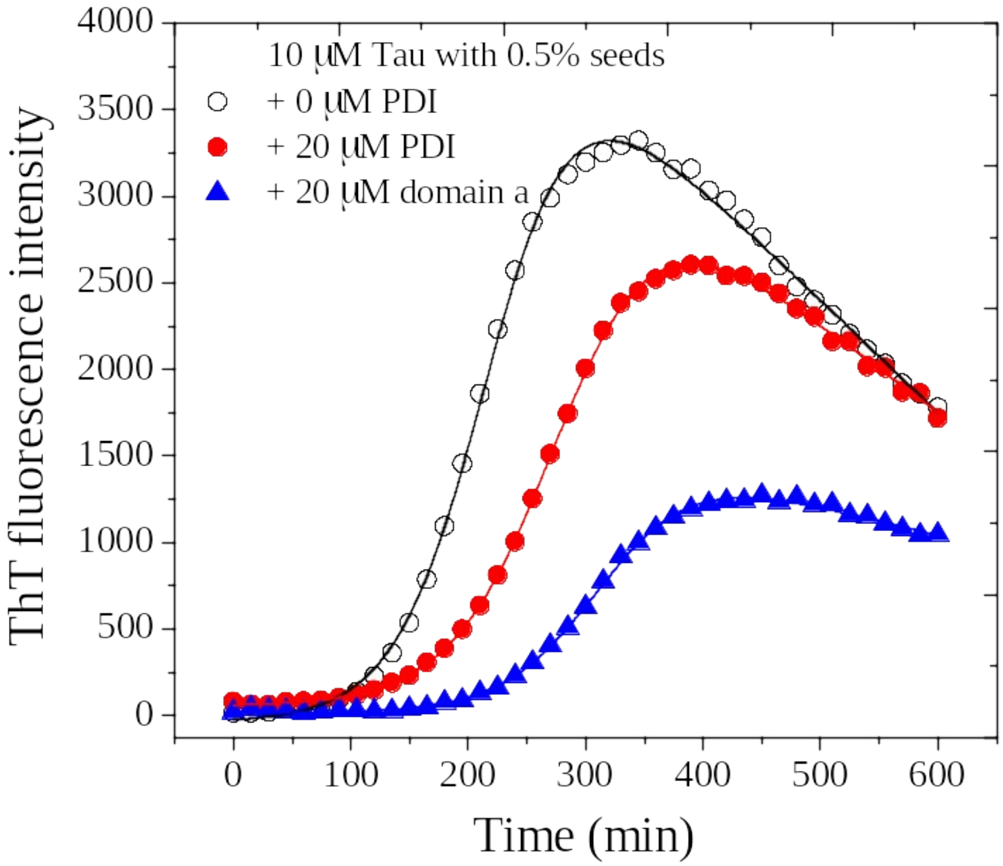
10 μM Tau with 0.5% seeds
+ 0 μM PDI
+ 20 μM PDI
+ 20 μM domain a
ThT fluorescence intensity
Time (min)
0
500
1000
1500
2000
2500
3000
3500
4000
0
100
200
300
400
500
600
