# Supplementary material for: Protein Disulfide Isomerase Interacts with Tau Protein and Inhibits Its Fibrillization
Source: PLoS One. 2013 Oct 2;8(10):e76657. doi: 10.1371/journal.pone.0076657 (PMC3788760; doi:10.1371/journal.pone.0076657)
Supplement: Supplementary file 5 [file pone.0076657.s005.doc]

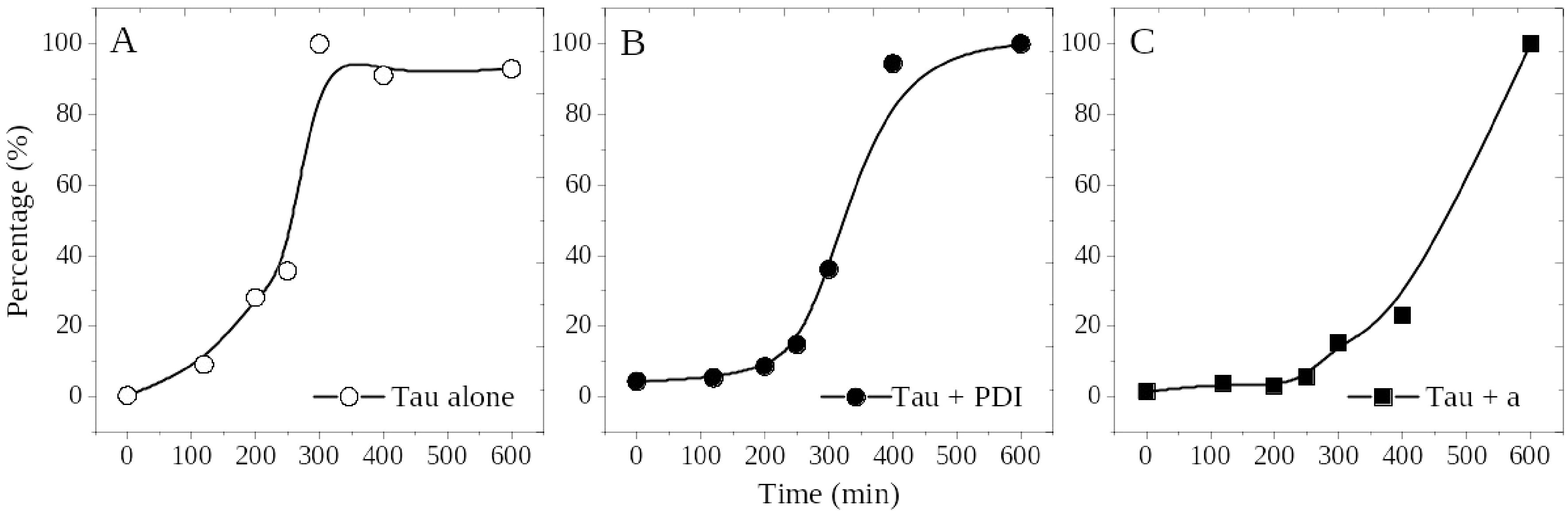
A
B
C
Percentage (%)
Time (min)
Tau alone
Tau + PDI
Tau + a
0
20
40
60
80
100
0
100
200
300
400
500
600
